# Reply to the ‘Comment on “The oxidation state in low-valent beryllium and magnesium compounds”’ by S. Pan and G. Frenking, *Chem. Sci.*, 2022, **13**, DOI: 10.1039/D2SC04231B

**DOI:** 10.1039/d2sc05769g

**Published:** 2022-12-15

**Authors:** Martí Gimferrer, Sergi Danés, Eva Vos, Cem B. Yildiz, Inés Corral, Anukul Jana, Pedro Salvador, Diego M. Andrada

**Affiliations:** a Departament de Química, Institut de Química Computacional i Catàlisi, Universitat de Girona c/M. Aurelia Capmany 69 17003 Girona Spain pedro.salvador@udg.edu; b General and Inorganic Chemistry Department, University of Saarland Campus C4.1 66123 Saarbruecken Germany diego.andrada@uni-saarland.de; c Departamento de Química, Universidad Autónoma de Madrid C/Francisco Tomás y Valiente 7 28049 Cantoblanco Madrid Spain ines.corral@uam.es; d Department of Medicinal and Aromatic Plants, Aksaray University Hacilar Harmani 2 68100 Aksaray Turkey; e Tata Institute of Fundamental Research Hyderabad Gopanpally 500046 Hyderabad Telangana India ajana@tifrh.res.in

## Abstract

A recent article by Pan and Frenking challenges our assignment of the oxidation state of low valent group 2 compounds. With this reply, we show that our assignment of Be(+2) and Mg(+2) oxidation states in Be(cAAC^Dip^)_2_ and Mg(cAAC^Dip^)_2_ is fully consistent with our data. Some of the arguments exposed by Pan and Frenking were based on visual inspection of our figures, rather than a thorough numerical analysis. We discuss with numerical proof that some of the statements made by the authors concerning our reported data are erroneous. In addition, we provide further evidence that the criterion of the lowest orbital interaction energy in the energy decomposition analysis (EDA) method is unsuitable as a general tool to assess the valence state of the fragments. Other indicators based on natural orbitals for chemical valence (NOCV) deliver a more reliable bonding picture. We also emphasize the importance of using stable wavefunctions for any kind of analysis, including EDA.

## Abstract 2

This work discusses on the correct assignment of the oxidation state in low-valent group 2 compounds. Additionally, we elaborate on common pitfalls describing the correct wavefunction and its implications.

## Graphical abstract

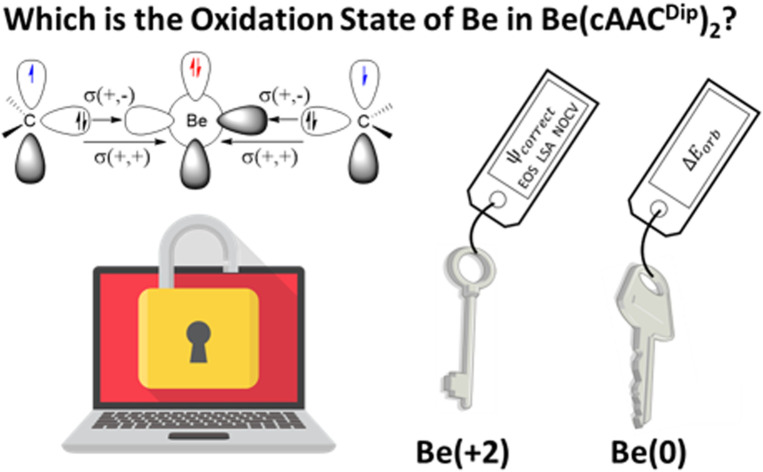

Pan and Frenking^1^ have challenged our recent assignment of the formal oxidation state of the beryllium and magnesium family of di-coordinated compounds ML_2_ (M = Be, Mg) with ligands of cyclic (alkyl) (amino) carbene (cAAC) and *N*-heterocyclic carbene (NHC) types.^2^ In a landmark study, the group of Braunschweig isolated and characterized the Be(cAAC^Dip^)_2_ complex.^3^ The authors described the system as the first example of a stable beryllium compound in the zero oxidation state Be(0), where the bonding situation consists of two donor–acceptor interactions between cAAC ligands and the central atom cAAC(0) ⇆ Be(0) ⇄ cAAC(0). The preparation of the heavier analogue Mg(cAAC^Dip^)_2_ has been attempted by Turner, but under the reaction conditions a ligand activation is observed, giving evidence for a highly reactive transient species.^4^ In our original work,^2^ we provided an alternative picture where the strong π-acidity of the ligand causes its own reduction giving rise to the singlet diradicaloid L(−1) → E(+2) ← L(−1) situation.^2^ The non-innocent behavior of cAAC ligands in main group and transition metal complexes has been discussed in a number of computational and experimental studies.^5–9^

One of the most relevant findings of our work^2^ is that the closed-shell single determinant wavefunction is either not stable (B3LYP) or higher in energy than a broken-symmetry KS-DFT solution. This indication of significant static correlation prompted us to rely on multireference wavefunctions (CASSCF in particular) to study the electronic structure of these species. Besides the usual electronic structure indicators, we applied the effective oxidation state (EOS) analysis to assign the oxidation state of the central atom and that of the ligands. This approach, introduced in 2015 by some of us,^10^ has already been tested on a wide variety of challenging systems, exposing some limitations of algorithms preferred by IUPAC.^11^ In contrast to energy decomposition analysis (EDA),^12–14^ EOS was specifically devised solely for the purpose of assigning oxidation states. It should be noted that other approaches specifically designed for OS assignment such as Head-Gordon's Localized Orbital Bonding Analysis (LOBA)^15^ or the recently introduced Oxidation State Localized Orbitals (OSLO)^16^ can only be applied for single-determinant wavefunctions. EOS is the only scheme of that kind that can be applied on equal footing for single-determinant and multiconfigurational wavefunctions, and hence it was the natural choice for our study.

Remarkably, only a small part of the criticism of Pan and Frenking actually refers to our preferred methodological framework, with arguments that are rather bizarre or simply faulty. For instance, they state that the EOS scheme “[…] is based on atomic fragments that are defined in different ways and can lead to different results”.^1^ While this is a pretty accurate description of the EDA approach, it does not apply to EOS analysis. In EOS, one needs to define the composing fragments, in this case the central atom and each ligand, but not their reference state. As such, the EOS approach is free of bias (*vide infra*).^6^ Then, the occupation of the fragment's domain natural orbitals (*i.e.* Mayer's effective fragment orbitals,^17^ henceforth EFOs) is used to assign electron pairs (or individual electrons in open-shell systems) to the fragments. Rather than rounding the occupation numbers to the closest integer, the EFOs of all fragments are sorted in descending order. The most plausible OS assignment (for all fragments) is the one in which the electron pairs are assigned to the highest occupied EFOs. A reliability index *R* (%) quantifies the extent to which the discrete OS values match the actual electronic structure. It is simply derived from the occupation numbers of the frontier EFOs in the EOS procedure.^10^

In fact, it is not the way the fragments are defined, but rather the identification of the atom in the molecule (AIM) that influences the numerical results to some extent. In Tables S9 and S10 of our original work, we already provided numerical evidence of the effect of using one or another AIM definition to obtain the EFOs.^2^ Pan and Frenking pinpoint the case of Be(cAAC^Me^)_2_, for which using classical Löwdin analysis results in a Be(0) OS (with a very low *R* (%) value, a close-call situation, in contrast with a clear Be(+2) OS when using more reliable AIM approaches like the Quantum Theory of Atoms in Molecules (QTAIM)^18^ or the Natural Atomic Orbital (NAO) basis.^19^ Pan and Frenking failed to notice that Löwdin's partial atomic charge on Be is −0.47 *e*, in sharp contrast with the other AIM approaches tested (well over +1.0 *e*). They also missed the case of Mg(cAAC^Me^)_2_. Here, the Löwdin partial charge on Mg is as low as −0.75 *e*, while it is *ca.* +0.4 *e* when using NAO or QTAIM. Remarkably, in all three cases the EOS analysis still yields the same undisputable OS assignment of Mg(0). A less biased perception of Tables S9 and S10 indicates that (i) EOS analysis is significantly more robust than partial atomic charges and (ii) partial atomic charges neither correspond to nor correlate with oxidation states, as repeatedly observed, and in line with IUPAC's definition of OS and the winner-takes-all principle.^20^

At this point it should become clear that we did not “omit the possibility that the metal atoms has the oxidation state +1”: such a faddy result (which necessarily implies a formal OS of −½ for each of the ligands) simply cannot stem from the wavefunction analysis. Artificially forcing the EOS scheme to produce a particular set of OS on the fragments, by definition, results in a lower *R* (%) value (in fact, even below the worst-case scenario of *R* (%) = 50).

Pan and Frenking also cast doubts on the fact that for Mg(cAAC^Me^)_2_ we obtain Mg(0) while for Mg(cAAC^Dip^)_2_ it is Mg(+2). Accordingly, “a drastic alteration in the nature of the Mg-cAAC^R^ bond from Mg(0) to Mg(2+) by changing the rather remote substituent R is questionable”. They regrettably failed to notice in Table 1 and Fig. 1 of our manuscript that the C–Mg–C bond angle goes from 107.8° to almost collinear (179°) by the steric effect of the remote substituent.

**Table 1:** EDA-NOCV of Be(cAAC^Dip^)_2_ at the B3LYP-D3(BJ)/TZ2P level of theory. The lowest Δ*E*_orb-corr_ is highlighted in bold. Energy values are given in kcal mol^−1^

	Be^0^(^1^D, 2s^0^2p_⊥_^2^); (cAAC)_2_ (singlet)	Be^+^(^2^P, 2s^0^2p_⊥_^1^); (cAAC)_2_^1−^ (doublet)	Be^2+^(^1^S, 2s^0^2p_⊥_^0^); (cAAC)_2_^2−^ (singlet)
Δ*E*_int_	−287.1	−426.0	−847.9
Δ*E*_Pauli_	157.4	99.9	105.8
Δ*E*_disp_^a^	−10.5 (2.4%)	−10.5 (2.0%)	−10.5 (1.1%)
Δ*E*_elstat_^a^	−202.6 (45.6%)	−276.3 (52.5%)	−499.4 (52.4%)
Δ*E*_orb_	−231.4	−239.3	−401.4
Δ*E*_orb-HF_	0.0	0.2	−42.4
Δ*E*_orb-corr_^a^	**−231.4** (52.1%)	−239.1 (45.5%)	−443.8 (46.5%)
Δ*E*_orb-σ(+,+)_^b^	−18.3 (7.9%)	−28.6 (12.4%)	−45.7 (10.3%)
Δ*E*_orb-σ(+,−)_^b^	−51.9 (22.4%)	−68.6 (28.7%)	−90.3 (20.3%)
Δ*E*_orb-π_^b^	−150.7 (65.1%)	−106.2 (44.4%)	−211.6 (47.7%)
Δ*E*_orb-rest_^b^	−10.4 (4.5%)	−34.6 (14.5%)	−53.9 (12.1%)
〈*S*〉^2^	0.571		

a The value in parentheses gives the percentage contribution to the total attractive interactions Δ*E*_elstat_ + Δ*E*_orb-corr_ + Δ*E*_disp_.

b The values in parentheses give the percentage contribution to the total orbital interaction Δ*E*_orb-corr_.

**Fig. 1:**
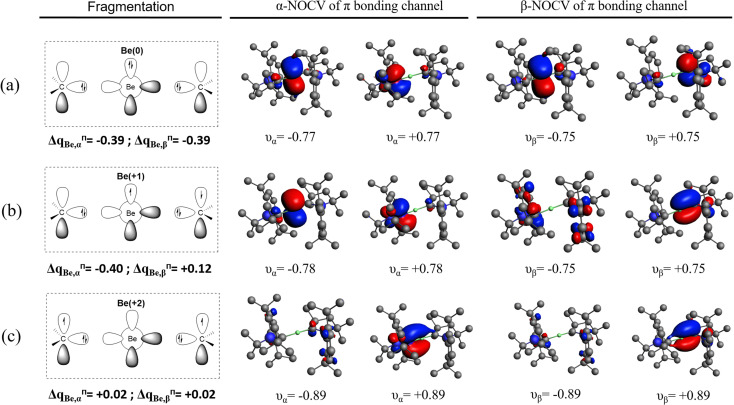
NOCV Orbitals (isocontour = 0.04) of the π bonding channel for Be(cAAC^Dip^)_2_ with fragments (a) Be^0^(^1^D, 2s^0^2p^2^) and (cAAC^Dip^)_2_, (b) Be^+^(^2^P, 2s^0^2p^1^) and (cAAC^Dip^)_2_^1−^ and (c) Be^2+^(^1^S, 2s^0^2p^0^) and (cAAC^Dip^)_2_^2−^.

Finally, we must also refer to Pan and Frenking most unjustified criticism: “[…] the reported bond dissociation energies (BDEs) given in Tables S5–S7 of the ESI of their work show very similar BDE values at different levels of theory, which question the value of the *R* (%) data”. In fact, the *R* (%) values are obtained from the EOS analysis of the multireference CASSCF wavefunctions. There is no relationship between the BDEs and the *R* (%).

Pan and Frenking also confound some statements and data we provide in the introductory part of our original manuscript, which merely served as hints to illustrate that the original Be(0) assignment might not be appropriate. This background information was not implying that Be(0) assignment must be incorrect, but was rather highlighting the uncertainty in previous assessment that prompted the more comprehensive analysis we performed in our work.

From their comments it appears that we actually share the view on most of the fundamental aspects of the oxidation state concept, but again, they distorted our statements. First of all, we agree with Pan and Frenking that merely considering the atomic electronegativities of the contact atoms to decide the fate of the electrons of a bond is troublesome. Some of us have already discussed this issue in detail,^10^ and that is precisely why we advocate for alternative approaches to simplistic IUPAC algorithms, such as EOS.

Now, we did not question the existence of dative bonds between the ligands and Be/Mg on the basis of their respective electronegativities as Pan and Frenking insinuated. This is clearly not the point. The point is, in the context of IUPAC's ionic approximation (IA), any bond (dative or covalent) will have the corresponding electron pair assigned to either Be/Mg or C, and in the most straightforward application of IA, it should be assigned to the most electronegative atom,^20^ which is the carbon atom. This is what stems from EOS analysis in the cases in question such as Be(cAAC^Dip^)_2_, as illustrated in Fig. S37 of our original manuscript. The occupation of the σ-type EFO on the ligands (0.85) is much higher than that of the s-type EFO on Be (0.18), which results in the electron pair of the σ M–L bond unambiguously assigned to the ligands.

What is really relevant from the OS point of view is the assignment of the π electrons of the system. In the Be(0) picture, the electrons of the 3c–2e π-bond are assigned to the less electronegative atom, at odds with IUPAC's simple rules.^20^ Since the latter are not foolproof we conducted EOS analysis, which in this case is crystal clear: as shown in Fig. S37 of ref. 2, the occupation of the p-type EFO on Be (0.12) is much smaller that of the π-type EFO of the ligands (0.44), which keep the electrons, resulting in a Be(+2) picture.

In our original manuscript, we also explicitly stated that Ponec and Cooper had also questioned the fate of the π-electrons using domain-averaged Fermi-hole (DAFH) analysis for a Be(cAAC^H^)_2_ model system.^21^ According to Ponec and Cooper, the electron pair formally originally occupying the Be(2p_π_) orbital contributes symmetrically to such an extent into the originally empty π orbitals on the carbene-like moieties that there is only a very small residual population on Be. In fact, the DAFH analysis reveals a contribution to the π bonding of 0.14 from Be and 0.95 from each of the ligands (see Fig. 1 of ref. ^21^). This is in full agreement with our results and with a Be(+2) formal valence state.

Pan and Frenking also appear to estimate the contribution of an AO to a given MO just by looking at the orbital plot. They focus on a π-type natural orbital with a natural occupation of 1.80. They claim without any proof that the largest coefficient of that orbital is at the Be atom. Then, according to IUPAC's IA, the electron pair should be assigned to Be. They quote a statement from ref. 20 that in our view represents a naïve and ambiguous point on the relationship between AO coefficients and OS. This issue has already been discussed elsewhere by some of us,^22^ so we will only provide a quick answer to Pan and Frenking. First of all, the orbital they refer is a natural orbital corresponding to our CASSCF description of Be(NHC^Dip^)_2_, not Be(cAAC^Dip^)_2_. For the latter, the relevant orbital is given in Fig. S15 of our original manuscript,^2^ with a natural occupation of 1.64. It is not so obvious which is the contribution of a given fragment holding a set of non-orthogonal AOs to a molecular or natural orbital. One may consider an orthogonal basis instead, such as a Löwdin's variant implemented in pySCF.^23^ The orbital coefficients of the 2p_*z*_ and 3p_*z*_ AOs of Be are 0.399 and −0.096 for Be(NHC^Dip^)_2_, and 0.291 and −0.095 for Be(cAAC^Dip^)_2_. For comparison, just the coefficient of the 2p_*z*_ AOs of each of the two contact carbon atoms of the ligands is 2 × 0.495 and 0.541 and 0.512 (non-symmetric orbital), respectively. The population of these natural orbitals on Be (*i.e.* sum of the square of the coefficients on an orthogonal basis) is merely 0.18 and 0.11, respectively, not too different from the DAFH and EFO occupations discussed above. Looks can be deceiving.

We do concede to Pan and Frenking that the excitation energies of Mg that we discussed in the Introduction of ref. ^2^ are plainly wrong. That was a regrettable mistake and we are grateful to them for pointing it out, despite being irrelevant to the discussion. Pan and Frenking used the ionization potential and electron affinity of the fragments. They took the electron affinity of cAAC^Dip^ from a PBE0/def2-TZVP calculation without Zero Point Vibration Energy (ZPVE) correction,^24^ which does not meet the definition of molecular electron affinity^25^ (it is not 18.4 kcal mol^−1^ but 14.5 kcal mol^−1^). Surely, the ionization energies required to achieve a real (not formal!) Be^2+^ species are much higher than those needed to reorganize the valence electron in a Be(0) picture, but this does not preclude the possibility of a formal Be(+2) OS. Otherwise, how to justify the O(−2) formal OS in H_2_O, where the formation of O^2−^ (which is unstable, by the way) from O(0) is endothermic by +167.8 kcal mol^−1^ (ref. 26 and 27) and the ionization potential of H is +313.3 kcal mol^−1^?^28^ Surely, Pan and Frenking are not challenging the formal OS assignments in the water molecule.

Another set of criticism from Pan and Frenking points towards our illustrative EDA calculations and EDA-NOCV analysis of these Be and Mg complexes, reported in the ESI of our original work.^2^ Pan and Frenking stated: “we repeated the calculations at the same level of theory as the authors but with inclusion of dispersion interactions, which had not been considered. We found that the Δ*E*_orb_ values using singly charged and neutral fragments become nearly equal, which means that Be(cAAC^Dip^) may be described with the oxidation states Be(0) or Be(+1) but clearly not with Be(+2).” Unfortunately, these calculations were neither reported nor used in their comment, and instead they reused our original Table S8 as Table 1 in their comment, which in fact always included dispersion corrections. First of all, assuming they did not reoptimize the geometry, the inclusion of dispersion will only provide a new stabilizing term Δ*E*_disp_, with exactly the same Δ*E*_orb_ values. Secondly, they complain about our notation, where the total orbital interaction term Δ*E*_orb-corr_ is presented as a sum of Δ*E*_orb_ and Δ*E*_orb-HF_ contributions, and they erroneously assume that we used them for our interpretation. Taking into account that the analysis is performed at the B3LYP-D3(BJ)/TZ2P level of theory, the latter corresponds to the contribution from 20% of exact-exchange of the density functional approximation. The reason the two terms are given explicitly is connected to the fact that the current implementation of EDA-NOCV does not decompose the non-local HF-like exchange contribution to Δ*E*_orb_.^29^ Such information has been described in dedicated technical literature and also could be easily checked in the outputs of their own calculations, or even in former tables reported by Pan and Frenking.^30,31^ Finally, and more importantly, Pan and Frenking claim that our analysis of the electron flow of the NOCVs is wrong just by how the deformation density looks like in our Fig. S41. They ignore the actual contributions of the symmetry-adapted fragment orbital (SFO) basis to the deformation density, which are also provided in our Fig. 1.

Let us repeat here our point concerning the π channel from the NOCV analysis. For the sake of simplicity, we will consider the total *α* and *β* contributions together. For each channel *k*, the deformation density is written as1
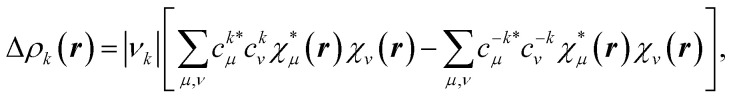
where *ν*_*k*_ is the eigenvalue (in absolute value) and the pair of NOCVs are expressed in the (orthogonal) SFO basis {*χ*} by the coefficients *c*^*k*^. Integrating the deformation density leads to2
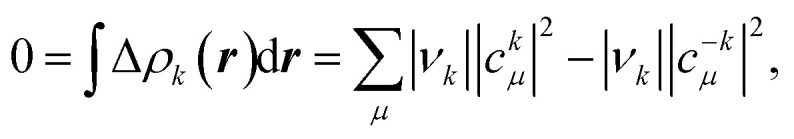
which readily affords a decomposition in terms of the SFO basis. The most significant contributions of the SFOs can be found in the output of a typical NOCV run. When using a Be^0^ reference, the corresponding eigenvalue is *ca.* |1.5|, meaning that an electron flow of 1.5 *e* is associated with this particular channel of the deformation density. In this case, it can be seen that the 2p_*z*_-type SFO on Be exhibits a very large and negative contribution of −1.49 *e*, meaning that electrons move from this orbital to those with positive contribution (ligands, in this case). This SFO contribution essentially fully accounts for the whole charge transfer of this channel. When using a Be^2+^ reference, the eigenvalue of the deformation density is |1.8|, but the contribution of the 2p_*z*_-type SFO on Be is merely +0.42 *e*. The positive contributions from other SFOs of the Be moiety are negligible, so *ca.* necessarily +1.4 *e* move into the ligand (and from the ligands, as indicated by the ligand-centered larger negative SFO contributions). Regrettably, Pan and Frenking strongly relied on our selected isocontour value (our Fig. S41 of the original manuscript and Fig. 2 in their comment) although the orbital interaction shows a Be contribution only at low isocontour values, while for bigger isocontour values it shows only the participation of the carbene carbon of the cAAC ligands (Fig. 2). This intrafragment charge-transfer process can be even more clearly seen with the NOCV orbitals, where the major contribution comes from the π-orbital of the cAAC ligands (Fig. 1).

**Fig. 2:**
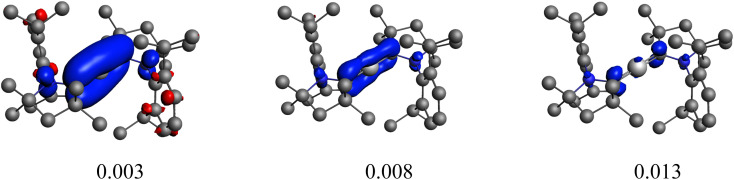
Plot of the main deformation density (Δ*ρ*) at different isocontour values of the pairwise orbital interactions between Be^2+^(^1^S, 2s^0^2p^0^) and (cAAC^Dip^)_2_^2−^ within Be(cAAC^Dip^)_2_. The red colour shows the charge outflow, whereas blue shows charge density accumulation.

A similar analysis can now be carried out not in the orbital space but in the real space, using a Hirshfeld-type partitioning for the entire fragments (*A*),^32^3
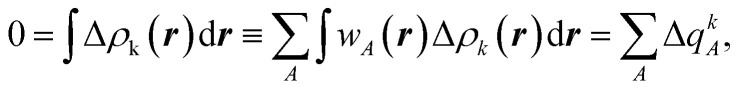
which affords the same picture albeit with a much smaller charge transfer (−0.78 *e* on Be for the Be^0^ reference and +0.04 *e* on Be for the Be^2+^ reference) (Fig. 1). Hence, the actual numbers, and not a mere picture, evidence that there is much less electron flow between fragments using the Be^2+^ reference, thus invalidating the Pan and Frenking claim.

For completeness, we have performed the EDA-NOCV analysis with the correct wavefunction also for the rather odd Be^1+^ fragmentation (see Table 1). The NOCVs of the π-bonding channel using a Be^1+^ fragmentation indicate an intermediate situation between using Be^2+^ and Be^0^, as might be anticipated. The eigenvalue of the deformation density is |1.54|, while the contribution of the 2p_*z*_-type SFO on Be is −0.51 *e*, slightly larger than the value using Be^2+^ discussed above (+0.42 *e*). In the Be^1+^ case, however, it originates from a partial cancelation of *α* (−0.75 *e*) and *β* (+0.24 *e*) contributions, thus indicating a much significant charge-reorganization than when using Be^2+^ as reference. Yet, from an energy perspective, Be^0^ and Be^1+^ show lower Δ*E*_orb_ values than the Be^2+^ fragmentation scheme. Does this necessarily mean that the oxidation state of Be cannot be Be(+2), while all other evidence clearly points toward this situation? Has the criterion of the lowest orbital interaction term been carefully tested in the past? In this sense, some of us considered the simplest case of H_2_O when evaluating an OS assignment method based on the position of the centroids of localized orbitals with respect to the atoms.^33^ We found that the method failed to describe H_2_O as formally H(+1) and O(−2). As will become clear later on, this kind of proof of concept calculations were regrettably skipped when putting forward EDA's orbital interaction energy as a general criterion to establish appropriate valence states and OS (*vide infra*).

**Table 2:** EDA-NOCV results of Ca–CO_2_ at the B3LYP-D3(BJ)/TZ2P level of theory with OSS and CSS wavefunctions. Energy values in kcal mol^−1^. The fragments are Ca^+^(4s^1^) and CO_2_^−^(D)

	OSS WF	CSS WF
Δ*E*_int_	−171.4	−168.2
Δ*E*_Pauli_	108.4	108.4
Δ*E*_elect_^a^	−206.4 (73.7%)	−206.4 (74.6%)
Δ*E*_disp_^a^	−1.90 (0.68%)	−1.89 (0.68%)
Δ*E*_orb_	−71.6	−68.3
Δ*E*_HF-corr_	0.00	0.00
Δ*E*_orb-corr_^a^	−71.6 (25.6%)	−68.3 (24.7%)
Δ*E*_orb-ρ1_^b^	−26.9 (37.6%)	−25.4 (37.2%)
Δ*E*_orb-ρ2_^b^	−18.8 (26.3%)	−19.3 (28.3%)
Δ*E*_orb-ρ3_^b^	−11.5 (16.1%)	−9.0 (13.2%)
Δ*E*_orb-ρ4_^b^	−5.2 (7.3%)	−5.6 (8.2%)
Δ*E*_orb-ρ5_^b^	−3.7 (5.2%)	−3.9 (5.7%)
Δ*E*_orb-rest_^b^	−5.5 (7.7%)	−5.1 (7.5%)

a The value in parentheses gives the percentage contribution to the total attractive interactions Δ*E*_elstat_ + Δ*E*_orb-corr_ + Δ*E*_disp_.

b The values in parentheses give the percentage contribution to the total orbital interaction Δ*E*_orb-corr_.

After having addressed Pan and Frenking's inaccurate statements, let us go back to square one. As mentioned above, the closed-shell KS-DFT wavefunction of many of these systems are either not stable or a lower in energy broken-symmetry solution is found. In the case of Be(cAAC^Dip^)_2_, the corresponding 〈*S*^2^〉 value is 0.57, which cannot be ignored at all. Indeed, this is supported by the computed D1 diagnostic,^34^ which exceeds in all the cases the reference value. Consistently, the weight of the closed-shell configuration is for all the complexes below 0.85. Local spin analysis^35^ thus reveals a significant diradicaloid character, with local spins centered at both cAAC^Dip^ ligands. That is why we claim that these systems have diradicaloid character, not because they exhibit a small singlet–triplet gap, as Pan and Frenking stated. The CASSCF description also confirms this picture, both from the significant occupation of the antibonding natural orbital of the Be(cAAC^Dip^)_2_ π bond (0.36) and from the local spin analysis values reported in Table 1 of our original paper.^2^ This situation is quite similar to that of the largely discussed bonding in the [NaBH_3_]^−^ cluster.^36^ In our earlier work on that subject,^36^ we already showed that “EDA cannot distinguish an electron-sharing interaction from a spin-polarized one (diradicaloid)”, at least by only looking at the energy components. Pan and Frenking do not confront the statement and clearly underestimate the implications of this fact, by stating that these are subtle differences and fall outside the scope of EDA. We could not agree more with their own statement: “the EDA is as good as the quantum theoretical method on which it is based”.^1^ We in fact extend it to any wavefunction analysis method. That is precisely why one should make sure that an appropriate wavefunction is considered. At least one can check whether it has internal instabilities or not. But how can this not be relevant for OS assignment from wavefunction analysis? If a given bond between A and B is “electron-sharing”, the electron pair will be allocated to either A or B depending on the direction of the ionic approximation. If there is enough spin polarization of the bond, a more reasonable result might be the homolytic distribution of electrons,^37^ since in the extreme case (a pure diradical) there is no “sharing” of electrons. The structure of the single-determinant closed-shell wavefunction and that of a broken-symmetry one can be very different, and this can even be taken into consideration for the EDA analysis (Fig. 3). In contrast, they justify their systematic misuse of EDA with “[…] it is odd to criticize the method for not providing information about a particular property that is not the target of EDA.” Should we stop caring about using the correct wavefunction?

**Fig. 3:**
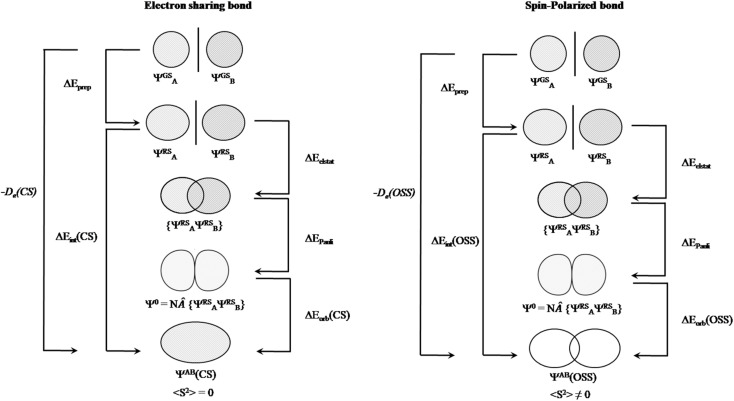
Energy decomposition analysis scheme for electron sharing (left) and spin-polarized bond (right) situation. Adapted from ref. 38.

Notably, Pan and Frenking brought the case of the Ca–CO_2_ complex in their comment,^39^ as an example where EDA-NOCV works for a system with open-shell singlet ground state character. Indeed, this is not their best example since one can clearly notice in their original paper that they have in fact used once more a closed-shell unstable wavefunction in their EDA-NOCV analysis, ignoring their own described diradical character.^39^ We have carried out the correct calculations, and we also present herein the obtained results. Table 2 gathers the outcome of the EDA for the closed-shell (unstable) and open-shell wavefunctions for the situation where the fragments are a Ca^+^(4s^1^) cation in the doublet ground state and a doublet ground state CO_2_^−^ anion. The results for both calculations are very similar: the only change in the energy components is in the Δ*E*_orb_, (*i.e.* 3.2 kcal mol^−1^ in the Δ*E*_int_), as illustrated in Fig. 3. This is because using closed-shell (unstable) or open-shell (stable) wavefunctions only affects the last step of the EDA process. Interestingly, the associated NOCVs exhibit significant differences in the charge transfer, as shown in Fig. 4. In the wrongly computed example by Pan and Frenking, the first deformation density channel has an eigenvalue of 1.26, with main contributions from Ca^+^(4s^1^) to the CO_2_^−^ SOMO (Fig. 4 CSS wavefunction). Note that their original article does not include this information.^39^ Applying EDA to the correct OSS wavefunction leads to a decrease of the eigenvalue to only 0.60, indicating a significant change in the electron density. Indeed, applying EOS to the closed-shell wavefunction indicates a Ca(+2) with *R* (%) = 94.4, while with the OSS wavefunction, EOS unambiguously indicates now an oxidation state Ca(+1) with *R* (%) = 93.6, which is the assignation Frenking reported by analyzing the wrong wavefunction.

**Fig. 4:**
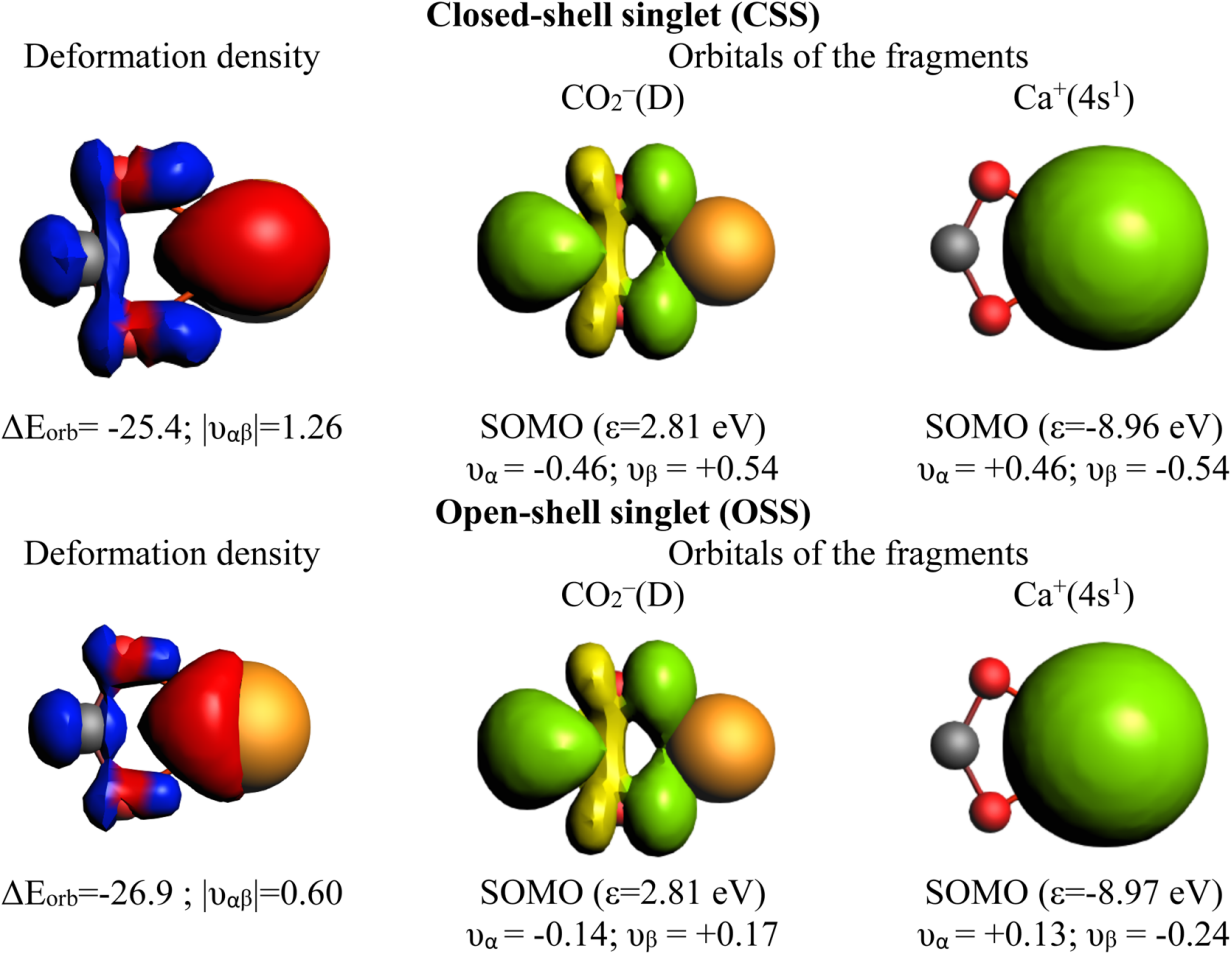
Plot of the main deformation density Δ*ρ*_1_ (isovalue = 0.003) of the pairwise orbital interaction and shape of the most important occupied and vacant orbitals (isovalue = 0.03) in the Ca–CO_2_ complex in the unstable closed-shell singlet (CSS) and the stable open-shell singlet (OSS) wavefunction with the orbital interaction energies Δ*E*_orb_ (in kcal mol^−1^) and the NOCV eigenvalues *ν* (in *e*). The fragments are Ca^+^(4s^1^) and CO_2_^−^(D). For the deformation densities, the direction of the charge flow is red → blue. The eigenvalues *ν* indicate the amount of donated (negative numbers) and accepted charge (positive numbers). The occupied orbitals are shown in yellow and green for the different phases, while the unoccupied orbitals are in light yellow and light green.

It is evident that Pan and Frenking are only stacked in the criterion of the lowest orbital interaction term, which looks more like a “credo” than a carefully tested indication of the best electronic reference situation. It is a good exercise to track back where their recited creed “those fragments which give the lowest energy change for the orbital interactions are the most suitable species to describe the bonding interaction” comes from. According to them, this is a proven fact; however, their citations only go to articles with the same phrase and no proof. To the best of our knowledge, the first mention of this was given in 2007,^40,41^ but there is no sign of a thorough systematic study of this matter, only a belief.^42–46^ One should not forget the memorable Feynman's quote: one must not verify an idea using the same data that suggested the idea in the first place. It is interesting to point out that some authors have already questioned its validity.^6,47^ Also, as repeatedly stated by the original developers of the EDA, the orbital interaction term includes both charge-transfer and polarization contributions, also within the fragments as shown here, which can certainly blur the analysis.^48–51^ Some of us have been diagnosing the problems associated with such an energetic criterion when it comes to OS assignment in general (notice that it is never considered in any of the IUPAC technical reports), and found that EDA-NOCV can still be a valuable tool if the interfragment electron flow is considered instead. It is out of the scope of the present response to discuss the details, which will be given elsewhere soon,^52^ but we can already disclose a quite surprising result. There is no need to use the exotic Be(cAAC^Dip^)_2_ system to observe that the orbital interaction is not a good indicator of the best electronic reference. As shown on Table 3, BeH_2_ would be in fact best described as Be(0) and H(0) by the lowest orbital interaction energy criterion. Are Pan and Frenking also challenging the oxidation state of BeH_2_?

**Table 3:** EDA-NOCV of BeH_2_ at the B3LYP-D3(BJ)/TZ2P level of theory. The lowest Δ*E*_orb-corr_ is highlighted in bold. Energy values are given in kcal mol^−1^

	Be^0^(^3^P, 2s^1^2p_∥_^1^); (H)_2_ (*σ*_g_^1^*σ*_u_^1^)	Be^+^(^2^S, 2s^1^2p_∥_^0^); (H)_2_^−^ (*σ*_g_^1^*σ*_u_^2^)	Be^+^(^2^P, 2s^0^2p_∥_^1^); (H)_2_^−^ (*σ*_g_^2^*σ*_u_^1^)	Be^2+^(^1^S, 2s^0^2p_∥_^0^); (H)_2_^2−^ (*σ*_g_^2^*σ*_u_^2^)
Δ*E*_int_	−217.5	−358.7	−410.5	−863.8
Δ*E*_Pauli_	7.1	7.7	61.5	79.2
Δ*E*_disp_^a^	−0.5 (0.2%)	−0.5 (0.1%)	−0.5 (0.1%)	−0.5 (0.1%)
Δ*E*_elstat_^a^	−69.4 (30.9%)	−193.0 (52.7%)	−224.1 (47.5%)	−678.9 (72.0%)
Δ*E*_orb_	−154.7	−173.0	−247.4	−263.8
Δ*E*_orb-HF_	0.0	−0.1	0.0	0.1
Δ*E*_orb-corr_^a^	**−154.7** (68.9%)	−173.0 (47.2%)	−247.4 (52.4%)	−263.7 (28.0%)
*D*_e_ (BeH_2_ → Be + 2H)	156.3			
*D*_e_ (BeH_2_ → Be^2+^ + 2H^−^)	754.0			

a The value in parentheses gives the percentage contribution to the total attractive interactions Δ*E*_elstat_ + Δ*E*_orb-corr_ + Δ*E*_disp_.

## Conclusions

Our assignment of Be(+2) and Mg(+2) oxidation states in Be(cAAC^Dip^)_2_ and Mg(cAAC^Dip^)_2_ is fully consistent with our data. Although Pan and Frenking have claimed a critical inspection of our data, their arguments are based on superficial visual inspection of our figures rather than a thorough numerical analysis. Additionally, we showcase their recent flawed application of EDA to diradicaloid species Ca–CO_2_. We provide evidence that the criterion of the lowest orbital interaction energy using EDA to determine the valence state of fragments is intrinsically unreliable, since it already fails for a simple system such as BeH_2_. As the oxidation state is a density based property, NOCV analysis can be a more reliable indicator of the best reference state. EDA-NOCV is without doubt a very useful method for bonding analysis, yet it should be used with appropriate care.

## Author contributions

M. G., S. D., E. V., and C. Y. performed the calculations. A. J., I. C., P. S., and D. M. A. acquired funding and contributed methodologies. M. G., S. D., P. S., and D. M. A. prepared the manuscript. All authors contributed to the interpretation of the computed data and the writing and editing of the manuscript.

## Conflicts of interest

The authors declare no conflict of interest.

## Supplementary Material
